# Pregnancy Outcomes Among Women With and Without Severe Acute Respiratory Syndrome Coronavirus 2 Infection

**DOI:** 10.1001/jamanetworkopen.2020.29256

**Published:** 2020-11-19

**Authors:** Emily H. Adhikari, Wilmer Moreno, Amanda C. Zofkie, Lorre MacDonald, Donald D. McIntire, Rebecca R. J. Collins, Catherine Y. Spong

**Affiliations:** 1 Department of Obstetrics and Gynecology, The University of Texas Southwestern Medical Center, Dallas; 2 Women and Infants Specialty Health, Parkland Health and Hospital System, Dallas, Texas

## Abstract

**Importance:**

Published data suggest that there are increased hospitalizations, placental abnormalities, and rare neonatal transmission among pregnant women with coronavirus disease 2019 (COVID-19).

**Objectives:**

To evaluate adverse outcomes associated with severe acute respiratory syndrome coronavirus 2 (SARS-CoV-2) infection in pregnancy and to describe clinical management, disease progression, hospital admission, placental abnormalities, and neonatal outcomes.

**Design, Setting, and Participants:**

This observational cohort study of maternal and neonatal outcomes among delivered women with and without SARS-CoV-2 during pregnancy was conducted from March 18 through August 22, 2020, at Parkland Health and Hospital System (Dallas, Texas), a high-volume prenatal clinic system and public maternity hospital with widespread access to SARS-CoV-2 testing in outpatient, emergency department, and inpatient settings. Women were included if they were tested for SARS-CoV-2 during pregnancy and delivered. For placental analysis, the pathologist was blinded to illness severity.

**Exposures:**

SARS-CoV-2 infection during pregnancy.

**Main Outcomes and Measures:**

The primary outcome was a composite of preterm birth, preeclampsia with severe features, or cesarean delivery for abnormal fetal heart rate among women delivered after 20 weeks of gestation. Maternal illness severity, neonatal infection, and placental abnormalities were described.

**Results:**

From March 18 through August 22, 2020, 3374 pregnant women (mean [SD] age, 27.6 [6] years) tested for SARS-CoV-2 were delivered, including 252 who tested positive for SARS-CoV-2 and 3122 who tested negative. The cohort included 2520 Hispanic (75%), 619 Black (18%), and 125 White (4%) women. There were no differences in age, parity, body mass index, or diabetes among women with or without SARS-CoV-2. SARS-CoV-2 positivity was more common among Hispanic women (230 [91%] positive vs 2290 [73%] negative; difference, 17.9%; 95% CI, 12.3%-23.5%; *P* < .001). There was no difference in the composite primary outcome (52 women [21%] vs 684 women [23%]; relative risk, 0.94; 95% CI, 0.73-1.21; *P* = .64). Early neonatal SARS-CoV-2 infection occurred in 6 of 188 tested infants (3%), primarily born to asymptomatic or mildly symptomatic women. There were no placental pathologic differences by illness severity. Maternal illness at initial presentation was asymptomatic or mild in 239 women (95%), and 6 of those women (3%) developed severe or critical illness. Fourteen women (6%) were hospitalized for the indication of COVID-19.

**Conclusions and Relevance:**

In a large, single-institution cohort study, SARS-CoV-2 infection during pregnancy was not associated with adverse pregnancy outcomes. Neonatal infection may be as high as 3% and may occur predominantly among asymptomatic or mildly symptomatic women. Placental abnormalities were not associated with disease severity, and hospitalization frequency was similar to rates among nonpregnant women.

## Key Points

**Question:**

In a large county health care system with access to inpatient and outpatient testing, is severe acute respiratory syndrome coronavirus 2 (SARS-CoV-2) infection associated with pregnancy outcomes, maternal illness severity, placental pathology, and neonatal infections?

**Findings:**

In this cohort study of 252 SARS-CoV-2–positive and 3122 negative pregnant women tested in outpatient and inpatient settings at a large county medical center, adverse pregnancy outcomes were similar, and neonatal infection occurred in 3% of infants, predominantly among infants born to asymptomatic or mildly symptomatic women. Placental abnormalities were not associated with disease severity, and the rate of hospitalization was similar to rates among nonpregnant women.

**Meaning:**

These findings suggest that SARS-CoV-2 infection in pregnancy is not associated with adverse pregnancy outcomes.

## Teaser

This cohort study evaluates the adverse outcomes associated with severe acute respiratory syndrome coronavirus 2 (SARS-CoV-2) infection among pregnant women and describes clinical management, disease progression, hospital admission, placental abnormalities, and neonatal outcomes.

## Introduction

By September 7, 2020, severe acute respiratory syndrome coronavirus 2 (SARS-CoV-2) had infected more than 27 million people worldwide.^1^ Data in pregnancy are still emerging. In June 2020, the Centers for Disease Control and Prevention (CDC) reported that hospitalization with coronavirus disease 2019 (COVID-19) occurred in 31.5% of pregnant women compared with 5.8% of nonpregnant women.^2^ However, there were no data on the indication for hospitalization. Severe and critical illness has been described in pregnancy^3,4,5,6^; less complete is our understanding of illness severity and hospital admission rates in a population with widespread inpatient and outpatient testing. We also have little understanding of rates and characteristics of early neonatal SARS-Cov-2 infection. Although preliminary evidence suggests that adverse pregnancy outcomes, such as preterm birth, preeclampsia, and cesarean delivery, are higher in women with confirmed SARS-CoV-2 infection during pregnancy, few large-scale studies have been conducted with sufficient power to evaluate risk for specific adverse outcomes.^7^ Abnormal placental findings have been reported with specific concerns for placental vasculopathy and inflammatory infiltrates.^8,9^ The aims of our study were to conduct a comprehensive evaluation of adverse pregnancy outcomes associated with SARS-CoV-2 infection in pregnancy and to describe clinical management, maternal disease severity and clinical progression, hospital admission, placental abnormalities, and neonatal outcomes at a high-volume, urban maternity care center with widespread access to SARS-CoV-2 testing.

## Methods

This is an observational cohort study of maternal and neonatal outcomes among women with and without SARS-CoV-2 diagnosed during pregnancy. This study was approved by the institutional review board at the University of Texas Southwestern Medical Center, and a waiver of informed consent was granted because the research involved minimal risk to the patients. We followed the Strengthening the Reporting of Observational Studies in Epidemiology (STROBE) reporting guideline for cohort studies.^10^

Women were included if they were tested for SARS-CoV-2 during pregnancy and delivered at Parkland Health and Hospital System, which serves the medically indigent women of Dallas County. Testing was performed for outpatient areas and, before May 14, 2020, for inpatient areas on the basis of symptoms (fever, cough, dyspnea, myalgia, loss of smell or taste, vomiting, diarrhea, or sore throat) or specific risk criteria. These included contact with a confirmed or suspected case, incarceration or group home setting, homelessness, outside hospital transfers, or unknown results from COVID-19 testing ordered from an outside clinic or facility. A universal SARS-Cov-2 testing protocol was implemented in the labor and delivery unit on May 14, 2020. Testing was on-site or via drive-through at 10 community-based prenatal clinics, and on-site in emergency department (ED) and inpatient units. Diagnosis was by reverse transcriptase–polymerase chain reaction detection of SARS-CoV-2 nucleic acid using either nasal or nasopharyngeal specimens using 1 of 4 different qualitative assays.^11,12,13,14,15^ Women presenting for care with external positive testing results were included without repeat testing.

### Management of SARS-CoV-2 Infection in Pregnancy

Symptomatic outpatients tested for SARS-CoV-2 infection were followed up via telemedicine virtual visits, with scripted evaluation of symptoms and protocol-based management including instructions for referral to the ED for worsening respiratory symptoms or obstetric concerns. Among women admitted to the hospital, the primary indication for hospitalization was recorded as either obstetric (non–COVID-19 related) or COVID-19 related. Both asymptomatic and symptomatic women were managed on the labor and delivery unit or hospital wards if respiratory status was stable and noncritical. Symptomatic postpartum and nonlaboring antepartum women with respiratory illness or oxygen requirement were managed in a dedicated negative pressure isolation ward (COVID unit).

For women with severe or critical COVID-19, delivery was considered for new or worsening oxygen requirement if they were at or near term. COVID-19 was not considered an indication for cesarean delivery except in cases of fetal heart tracing abnormalities that were not improved with adequate maternal respiratory support or worsening maternal respiratory status with anticipated need for intubation and immediate prone positioning, which precluded fetal monitoring.^16^ Urgent delivery among women with severe COVID-19 pneumonia occurred within a dedicated operating room in the COVID unit. Women with severe or critical illness received COVID-19 therapies according to National Institutes of Health (NIH) guidelines.^17^

Neonatal testing protocols included SARS-CoV-2 testing at 24 and 48 hours among infants born within 4 weeks of maternal SARS-CoV-2 diagnosis or when clinically indicated; admission to a neonatal intensive care unit isolation ward was routine for infants born to symptomatic mothers until maternal transmission-based precautions could be discontinued or until discharge. Remote video access to infant rooms was provided to all postpartum women admitted to the COVID unit.

### Maternal, Obstetric, and Neonatal Outcomes

We included all delivered women with available SARS-CoV-2 testing. Demographic and baseline characteristics were compared among women with and without SARS-CoV-2 diagnosed during pregnancy. Race and ethnicity were defined by the investigators and were obtained by medical record review to examine disparities in COVID-19 diagnosis. The primary outcome was a composite of preterm birth (iatrogenic or spontaneous), preeclampsia with severe features, or cesarean delivery for abnormal fetal indication among women delivered after 20 weeks of gestation. Secondary outcomes included preterm birth, preeclampsia with severe features, and cesarean delivery analyzed individually, as well as additional adverse maternal and neonatal outcomes. Among women with COVID-19, we further characterized maternal disease severity at presentation according to the NIH COVID-19 illness severity classification, including asymptomatic, mild, moderate, severe, or critical.^17^ We compared trimester of diagnosis, course of illness, hospitalization indication, and select delivery outcomes according to maternal illness severity. Among all delivered women diagnosed with SARS-CoV-2 during pregnancy, we evaluated trimester of diagnosis and neonatal SARS-CoV-2 results according to maternal illness severity. Detailed review of placental abnormalities from deliveries of women with SARS-CoV-2 infection was performed by a pathologist blinded to maternal illness severity and outcomes.

### Statistical Analysis

Univariate analyses were performed using the *t* test or Wilcoxon rank-sum test for continuous outcomes and the Pearson χ^2^ test for categorical outcomes. Mantel-Haenszel χ^2^ test was used to evaluate trends. The *P* values for all hypotheses were 2-sided, and statistical significance was set at *P* < .05. Effect sizes were presented as unadjusted odds ratios (ORs) because demographic differences were not thought to have a significant impact on the association of SARS-CoV-2 positivity with obstetric outcomes. Statistical analysis was performed using SAS statistical software version 9.4 (SAS Institute). Sample size was calculated on the basis of a 21% frequency of a composite outcome of preterm birth, preeclampsia with severe features, and cesarean delivery for abnormal fetal heart rate in the year 2019 at our institution. Using an estimated overall positivity rate of 7%, according to our local epidemiology, to detect a 50% increase in the composite outcome with a power of 80% and a type I error of 5%, a sample size of at least 2014 including 150 SARS-CoV-2–positive and 1864 SARS-CoV-2–negative women would be required.

## Results

From March 18 through August 22, 2020, 3374 pregnant women (mean [SD] age, 27.6 [6] years) were tested for SARS-CoV-2 infection and delivered (Figure), including 252 who were SARS-CoV-2 positive and 3122 who were SARS-CoV-2 negative. Among pregnant women with SARS-CoV-2 infection, diagnosis was made in labor and delivery units for 172 women (68%), outpatient clinics for 67 women (27%), outside testing sites for 7 women (3%), the ED for 4 women (2%), and inpatient units for 2 women (<1%). Excluding abortuses, obstetric outcomes were analyzed for 245 SARS-CoV-2–positive and 3035 SARS-CoV-2–negative women who delivered after 20 weeks of gestation.

**Figure.**
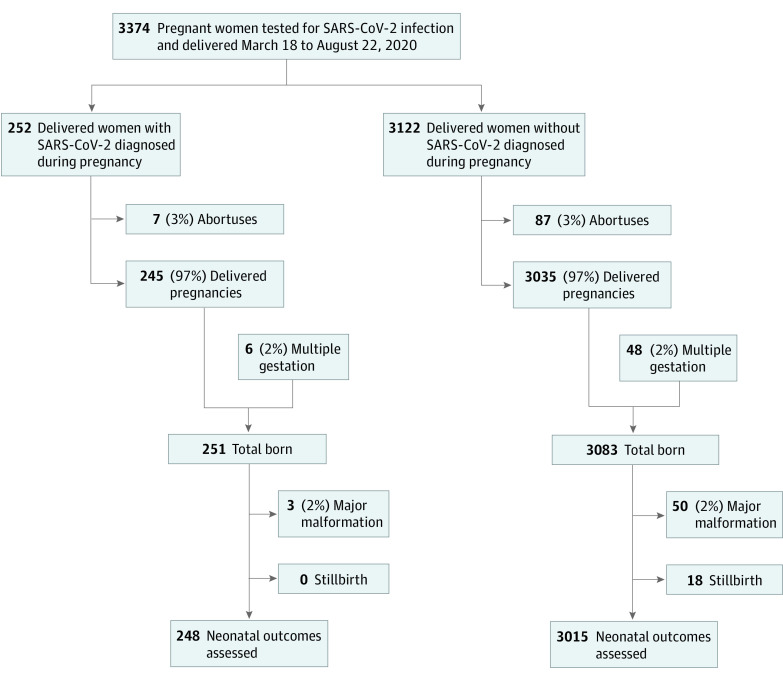
Flow Diagram of Pregnant Women Tested for Severe Acute Respiratory Syndrome Coronavirus 2 (SARS-CoV-2) Infection

The cohort included 2520 Hispanic women (75%), 619 non-Hispanic Black women (18%), 125 non-Hispanic White women (4%), and 110 women of other races/ethnicities (3%). SARS-CoV-2 positivity was most common among Hispanic women (230 [91%] positive vs 2290 [73%] negative; difference, 17.9%; 95% CI, 12.3%-23.5%; *P* < .001).There were no differences in maternal age, parity, body mass index, or diabetes among delivered women with and without SARS-CoV-2 (Table 1). There was no difference in the composite primary outcome of preterm birth, preeclampsia with severe features, and cesarean delivery for fetal indication among women with and without SARS-CoV-2 infection diagnosed during pregnancy (52 women [21%] vs 684 women [23%]; relative risk, 0.94; 95% CI, 0.73-1.21; *P* = .64) (Table 2). Among individual secondary outcomes, there were no differences. There were no stillbirths among women with SARS-CoV-2 during pregnancy.

**Table 1.** Demographic Characteristics Among Women With and Without SARS-CoV-2 Infection Diagnosed During Pregnancy

Characteristic	SARS-CoV-2 infection status, patients, No. (%)		*P* value
	Positive (n = 252)	Negative (n = 3122)	
Age, mean (SD), y	27.0 (6.6)	27.6 (6.4)	.11
Race			
Black, non-Hispanic	18 (7)	601 (19)	<.001^a^
White, non-Hispanic	2 (1)	123 (4)	
Hispanic	230 (91)	2290 (73)	
Other^b^	2 (1)	108 (3)	
Nulliparous	73 (29)	962 (31)	.54
Abortuses	7 (3)	87 (3)	.99
Body mass index, mean (SD)^c^	30.5 (7.2)	29.3 (6.6)	.01
Diabetes^d^	15 (6)	264 (8)	.17
Gestational	14 (6)	207 (7)	.51
Pregestational	1 (0.4)	57 (1.8)	.09
Multiple gestation^d^	6 (2)	48 (2)	.30
Chronic hypertension^d^	12 (5)	145 (5)	.93
Induction of labor^d^	65 (26)	770 (25)	.69

Abbreviation: SARS-CoV-2, severe acute respiratory syndrome coronavirus 2.

^a^ Denotes significant difference for all race groups.

^b^ Includes women who identified as non-Black and non-White race and Non-Hispanic ethnicity.

^c^ Body mass index is calculated as weight in kilograms divided by height in meters squared. Data are missing for 3 women who tested positive and for 73 women who tested negative.

^d^ Excludes abortuses.

**Table 2.** Obstetric and Neonatal Outcomes Among Delivered Women With and Without SARS-CoV-2 Infection Diagnosed During Pregnancy^a^^,^^b^

Characteristic	SARS-CoV-2 infection status, patients, No. (%)		*P* value	RR (95% CI)
	Positive (n = 245)	Negative (n = 3035)		
Primary outcome, composite^c^	52 (21)	684 (23)	.64	0.94 (0.73-1.21)
Secondary outcomes				
Gestational age <37 wk at delivery	27 (11)	328 (11)	.92	1.02 (0.70-1.48)
Categorical				
<34 wk	9 (4)	124 (4)	.42	
34 wk 0 d to 36 wk 6 d	18 (7)	204 (7)		
37 wk 0 d to 38 wk 6 d	75 (31)	819 (27)		
39 wk 0 d to 39 wk 6 d	81 (33)	949 (31)		
≥40 wk	62 (25)	939 (31)		
Preeclampsia with severe features	26 (11)	359 (12)	.57	0.90 (0.62-1.31)
Cesarean delivery	65 (27)	1011 (33)	.03	0.80 (0.64-0.99)
For abnormal fetal heart rate	7 (3)	153 (5)	.13	0.57 (0.27-1.20)
Spontaneous vaginal delivery	174 (71)	1975 (65)	.09	1.09 (1.00-1.19)
Abruption	0 (0.0)	24 (1)	.16	0.25 (0.02-4.14)
Clinical chorioamnionitis	24 (10)	345 (11)	.45	0.86 (0.58-1.28)
Stillbirth	0 (0)	18 (0.6)	.23	0.33 (0.02-5.48)
Excessive blood loss	17 (7)	278 (9)	.24	0.76 (0.47-1.21)
Transfusion	0 (0)	0 (0)	NA	NA
Anesthesia received				
Epidural	143 (58)	1846 (61)	.45	0.96 (0.86-1.07)
General endotracheal	2 (1)	53 (2)	.28	0.47 (0.11-1.91)
Neonates, No.^b^	248	3015		
Small for gestational age	31 (13)	316 (10)	.32	1.19 (0.84-1.68)
Meconium-stained amniotic fluid	53 (22)	571 (19)	.35	1.13 (0.88-1.45)
5-min Apgar score <4	0 (0)	37 (1.2)	.08	0.16 (0.01-2.63)
Umbilical cord blood pH <7.0^d^	1 (0.4)	9 (0.3)	.75	1.39 (0.18-10.94)
Mechanical ventilation in the first 24 h	0 (0)	14 (0.5)	.28	0.42 (0.03-6.99)
Required continuous positive airway pressure in the first 24 h	8 (3.3)	100 (3.3)	.94	0.97 (0.48-1.98)
Sepsis	0 (0)	6 (0.2)	.48	0.93 (0.05-16.52)

Abbreviations: NA, not applicable; RR, relative risk; SARS-CoV-2, severe acute respiratory syndrome coronavirus 2.

^a^ Excludes abortuses.

^b^ Includes multiple births but excludes stillbirths and major malformations as shown in the Figure.

^c^ Composite outcome of preterm birth (<37 weeks), preeclampsia with severe features, and cesarean delivery for abnormal fetal heart rate.

^d^ Data for pH are missing for 22 SARS-CoV-2–positive women and 184 SARS-CoV-2–negative women (*P* = .09 for missing pH).

### Neonatal Outcomes

Three infants with anomalies were born to women with SARs-CoV-2 (Figure). One infant had congenital pulmonary airway malformation diagnosed months before maternal infection, a second infant received a postnatal diagnosis of cleft palate and a hypoplastic thumb, and a third infant had characteristics of Down Syndrome. Among live-born infants without major malformations, the frequency of small for gestational age infants was not different (Table 2). The frequency of neonatal respiratory support required within 24 hours of birth did not differ.

### Maternal COVID-19 Illness Severity, Hospitalization, and Clinical Progression

Among 107 (42%) initially asymptomatic pregnant women diagnosed with SARS-CoV-2 infection, 98 (92%) remained asymptomatic, 7 (6%) developed mild symptoms, and 2 (2%) developed critical illness (Table 3). Among 132 women (52%) with initial mild illness, symptoms remained mild in 126 (95%), whereas 2 (2%) progressed to moderate illness and 4 (3%) progressed to severe illness. Among 10 women (4%) with initial moderate illness, 6 (60%) remained moderately ill, whereas 4 (40%) developed severe illness with oxygen requirement. Among 3 women (1%) with initial severe illness, 1 (33%) remained severely ill and 2 (67%) developed critical illness. Among all 239 women (95%) with asymptomatic or mild disease at initial presentation, 6 of those women (3%) subsequently developed severe or critical illness. Of the total cohort of 252 women, 13 (5%) presented with or developed severe or critical illness. There were no maternal deaths. Admission within 14 days for obstetric (non-COVID) indications occurred for 163 women (65%) with SARS-CoV-2 infection. In contrast, 14 (6%) of 252 SARS-CoV-2-positive pregnant women were hospitalized for management of COVID-19 pneumonia within 14 days of diagnosis or symptom onset. This included 1 woman (1%) with initial asymptomatic infection, 4 women (3%) with mild illness, 6 women (60%) with moderate illness, and 3 women (100%) with severe illness at initial presentation.

**Table 3.** Illness Severity, Progression, and Hospitalization Among Delivered Women Diagnosed With SARS-CoV-2 Infection During Pregnancy

COVID-19 illness severity at initial presentation	Patients, No. (%)							
	Total	Admitted within 14 d for obstetric indication^a^	Clinical progression among pregnant women diagnosed with SARS-CoV-2 infection					
			Asymptomatic	Mild	Moderate	Severe	Critical	Admitted within 14 d for COVID-19 pneumonia^a^
Asymptomatic	107 (42)	99 (93)	98 (92)	7 (6)	0	0	2 (2)	1 (1)^b^
Mild	132 (52)	62 (47)	NA	126 (95)	2 (2)	4 (3)	0	4 (3)
Moderate	10 (4)	2 (20)	NA	NA	6 (60)	4 (40)	0	6 (60)
Severe	3 (1)	0 (0)	NA	NA	NA	1 (33)	2 (67)	3 (100)
Critical	0	NA	NA	NA	NA	NA	NA	NA
Total	252	163 (65)	98 (39)	133 (53)	8 (3)	9 (4)	4 (2)	14 (6)

Abbreviations: COVID-19, coronavirus disease 2019; NA, not applicable; SARS-CoV-2, severe acute respiratory syndrome coronavirus 2.

^a^ Denotes admission within 14 days of symptom onset or diagnosis (if asymptomatic).

^b^ One asymptomatic woman developed critical COVID-19 illness while hospitalized for a non-COVID indication and is excluded from this group.

### Severe and Critical COVID-19 Pneumonia in Pregnancy

Thirteen women (5%) either presented with or developed severe or critical COVID-19 pneumonia. Respiratory support methods included low-flow nasal cannula for 7 women (54%), nonrebreather mask for 2 women (15%), high-flow nasal cannula for 2 women (15%), and mechanical ventilation for 2 women (15%). Venous thromboembolism was diagnosed in 1 woman (8%) treated with therapeutic anticoagulation. Intravenous remdesivir was administered for 5 women (38%), dexamethasone for 5 women (38%), convalescent plasma for 2 women (15%), and interleukin-6 inhibitor for 1 woman (8%). Other nonobstetric bacterial infections were treated in 3 women (23%). Among 13 women who presented with or developed severe or critical COVID-19 pneumonia, 2 (15%) received a diagnosis at less than 24 weeks’ gestation, including 1 who had a second trimester pregnancy loss during prolonged intubation and 1 who was discharged and returned to deliver spontaneously at 39 weeks. Eight women (62%) received a diagnosis between 24 and 37 weeks’ gestation, with 4 delivered while admitted (including 3 before 37 weeks) and 4 discharged after improvement (2 returning to deliver before 37 weeks). Among 3 women (23%) diagnosed at 37 weeks or greater, all were delivered during initial hospitalization (including 2 induced with subsequent vaginal delivery and 1 who underwent repeat cesarean delivery). Among 10 pregnant women with severe or critical illness diagnosed before 37 weeks, pregnancy loss or preterm birth (iatrogenic or spontaneous) occurred in 6 women (60%). When evaluating outcomes according to maternal illness severity, severe illness was significantly associated with either gestational or pregestational diabetes, and preterm delivery was significantly associated with increasing severity of maternal COVID-19 illness according to the NIH criteria (eTable 1 in the Supplement).

### Trimester of SARS-CoV-2 Diagnosis and Early Neonatal SARS-CoV-2 Infection

Among 245 women with SARS-CoV-2 during pregnancy who were delivered of live-born infants, 16 (7%) received a diagnosis during the second trimester and 227 (93%) received a diagnosis during the third trimester (Table 4). Among 188 tested neonates, 6 (3%) were positive for SARS-CoV-2 infection. All were born to women whose infection was diagnosed during the third trimester, including 4 women with asymptomatic, 1 with mild, and 1 with severe illness. Five infants (83%) were born vaginally, and 5 (83%) were born after 37 weeks. Among the 6 infants who tested positive, 4 were positive at 24 hours of life; 2 were negative at 24 hours and positive at 48 hours of life. Of the 4 infants who tested positive at 24 hours of life, repeat testing at 48 hours was performed for 3, and all remained positive. Although a specific route of SARS-CoV-2 transmission was not determined in most cases, intrauterine transmission was suspected for 1 preterm infant (34 weeks’ gestation) following vaginal delivery after prelabor rupture of membranes. This neonate had a mild febrile illness; placental examination revealed SARS-CoV-2 viral particles in the placental tissue by electron microscopy.^18^ This infant was born to a mother who initially presented with mild illness and subsequently developed pneumonia with hypoxia treated with oxygen by nasal cannula.

**Table 4.** Early Neonatal SARS-CoV-2 Infections by Trimester of Maternal Diagnosis and Maternal Illness Severity

Trimester of SARS-CoV-2 diagnosis	Infants with early neonatal SARS-CoV-2 infection, No.								
	Abortus	Live-born infants	Multiples	Maternal illness severity					
				Asymptomatic	Mild	Moderate	Severe	Critical	Total
First	4	0	0	NA	NA	NA	NA	NA	NA
Second	3	16	2	0/2 tested (3 live-born)	0/1 tested (13 live-born)	0	0 tested (1 live-born)	0/1 tested (1 live-born)	0/4 tested (18 live-born)
Third	0	229	4	4/90 tested (95 live-born)	1/82 tested (120 live-born)	0/4 tested (8 live-born)	1/6 tested (8 live-born)	0/2 tested (2 live-born)	6/184 tested (233 live-born)
Total	7	245	6	4/92 tested (98 live-born)	1/83 tested (133 live-born)	0/4 tested (8 live-born)	1/6 tested (9 live-born)	0/3 tested (3 live-born)	6/188 tested (251 live-born)

Abbreviations: NA, not applicable; SARS-CoV-2, severe acute respiratory syndrome coronavirus 2.

### Detailed Pathologic Analysis of Placentas

Among 187 available placentas, there were no differences in pathologic lesions stratified by maximum maternal illness severity except villous edema (eTable 2 in the Supplement). Of the 6 infants with early SARS-CoV-2 infection, 2 placentas were available for analysis, both with villous infarcts. The placenta from the preterm infant with intrauterine transmission demonstrated massive chronic intervillositis.

## Discussion

Women with SARS-CoV-2 infection were more likely to be of Hispanic ethnicity in our cohort. Although women of Hispanic ethnicity comprised 75% of current cohort, they accounted for more than 90% of SARS-CoV-2–positive women. Delivered women with SARS-CoV-2 infection during pregnancy did not have a significantly higher frequency of a composite primary outcome including preterm birth, preeclampsia with severe features, or cesarean delivery for abnormal fetal heart rate. There were no significant differences in other adverse pregnancy outcomes, although these comparisons were hypothesis generating. Maternal COVID-19 illness severity at initial presentation was asymptomatic or mild in 239 women (95%), and 6 of those women (3%) subsequently developed severe or critical COVID-19 pneumonia. Of the total cohort of 252 women, 13 (5%) presented with or developed severe or critical illness. Early neonatal SARS-CoV-2 occurred in 3% of tested infants, primarily among those born to asymptomatic or mildly symptomatic women. Among 10 pregnant women with severe or critical illness diagnosed before 37 weeks, pregnancy loss or preterm birth (iatrogenic or spontaneous) occurred in 6 women (60%). Although the risk of delivery at less than 37, 34, and 28 weeks increased with worsening maternal COVID-19 illness severity, no placental abnormalities were associated with illness severity.

The higher frequency of SARS-CoV-2 among Hispanic women in our study is consistent with data on racial and ethnic disparities in COVID-19 cases and deaths reported nationwide.^19^ Most studies in pregnancy published to date are case series^3,4,5,6,20,21^ comparing maternal or neonatal outcomes stratified by disease severity among women with confirmed COVID-19. In a recently published meta-analysis, Allotey et al^7^ reported higher preterm birth among pregnant women with COVID-19 (15.9%) compared with uninfected women (6.1%), although numbers included in that comparison were small and included case series, as well as preprints that had not been peer-reviewed. Our large, single-institution comparative analysis adds valuable information for counseling pregnant women with SARS-CoV-2 infection, the majority of whom will have mild symptoms but substantial anxiety.

In a descriptive series of 241 COVID-19–positive pregnant women, Khoury et al^4^ reported that 61% of women presenting to the labor and delivery unit were initially asymptomatic (compared with 42% in our cohort) and only 26% had mild symptoms (compared with 52% in our cohort). We believe the proportional differences in relative disease severity are related to the fact that our cohort includes all pregnant women tested in outpatient, ED, and inpatient settings. Outpatient SARS-CoV-2 testing was performed primarily for symptomatic women through drive-through or walk-up testing sites. High rates of outpatient test positivity combined with asymptomatic and mild infections diagnosed on labor and delivery reflected widespread community transmission.

In June 2020, the CDC reported that 31.5% of pregnant women with COVID-19 were hospitalized compared with 5.8% of nonpregnant women.^2^ There were no data on the indication for hospitalization. Our findings clarify that most women with asymptomatic or mild infection are admitted for obstetric indications. In our study, 14 (6%) of 252 women were hospitalized for the indication of COVID-19, similar to hospitalization frequency among nonpregnant women in the CDC report (5.8%).^2^ More recently, surveillance data published by the CDC again emphasized increased rates of COVID-19–related hospitalizations (41%), intensive care unit admission (16.2%), and mechanical ventilation (8.5%) among hospitalized pregnant women with symptomatic COVID-19.^22,23^ The major limitations in those reports are from the exclusion of pregnant women who receive their diagnoses in outpatient or ED settings and are never hospitalized. Our study includes these women in a comprehensive analysis and provides much needed evidence for counseling purposes.

Importantly, our findings that 5% of all delivered women with SARS-CoV-2 infection present with or develop severe or critical illness are novel and lower than rates in previous reports.^3,4,5,23,24^ Ascertainment of cases involved a coordinated effort among outpatient, ED, and inpatient staff to ensure that all women were managed with similar protocols developed by a dedicated COVID-19 care team. We suspect that the 5% rate of severe or critical COVID-19 illness in pregnancy reflects communities with widespread transmission, much of which may go undetected. Our findings that early neonatal infection rates may be as high as 3% and occur predominantly among asymptomatic or mildly symptomatic women adds needed context to published reports.

### Limitations

This study has limitations that should be addressed. It did not have power to detect differences in individual adverse outcomes; thus, conclusions made from these comparisons may not be generalizable. Although we were unable to test all women during the study period, we began testing symptomatic women soon after the first cases of local transmission were reported in Dallas County on March 12, 2020.^25^ There were no demographic differences in tested and untested women who delivered during the study period, minimizing potential selection bias. We did not include women admitted at other area hospitals and, thus, may have underdiagnosed some who developed severe or critical illness. Owing to test supply shortages, we could not use the same molecular test for all women. All positive tests, when associated with symptoms or representing the first positive test, were assumed to represent active infection; all negative tests were assumed to represent absence of infection. Not all neonates were tested for SARS-CoV-2; however, the risk of intrauterine infection in an otherwise healthy neonate born to a woman remote from her infection was thought to be extremely low. Furthermore, not all placentas were available for pathologic analysis.

## Conclusions

Further study is needed to understand whether maternal infection with SARS-CoV-2 is associated with long-term maternal or infant health. Except for a single case, in this cohort study, we were unable to determine whether diagnosis of early neonatal SARS-CoV-2 infection was the result of vertical or horizontal transmission, and little is known about specific risk factors for neonatal infection. Breastfeeding rates were not studied, and there is concern that separation of mother-infant dyads may impact breastfeeding and transferred immunity to the neonate. Finally, whether available COVID-19 therapeutic agents are effective in treating maternal illness or preventing neonatal infection is unclear, and inclusion of pregnant women in interventional trials is needed.

## Notes

Supplement. **eTable 1. **Characteristics and Delivery Outcomes Stratified by Illness Severity Among Women With SARS-CoV-2 During Pregnancy **eTable 2. **Pathologic Characteristics of Placentas From Deliveries Among Women With SARS-CoV-2 Infection During Pregnancy
