# Supplementary material for: Pregnancy Outcomes Among Women With and Without Severe Acute Respiratory Syndrome Coronavirus 2 Infection
Source: JAMA Netw Open. 2020 Nov 19;3(11):e2029256. doi: 10.1001/jamanetworkopen.2020.29256 (PMC7677755; doi:10.1001/jamanetworkopen.2020.29256)
Supplement: Supplement. — eTable 1. Characteristics and Delivery Outcomes Stratified by Illness Severity Among Women With SARS-CoV-2 During Pregnancy eTable 2. Pathologic Characteristics of Placentas From Deliveries Among Women With SARS-CoV-2 Infection During Pregnancy [file jamanetwopen-e2029256-s001.pdf]

Supplemental Online Content

Adhikari EH, Moreno W, Zofkie AC. Pregnancy outcomes among women with and without severe acute respiratory syndrome coronavirus 2 infection. *JAMA Netw Open*. 2020;3(11):e2029256. doi:10.1001/jamanetworkopen.2020.29256

eTable 1. Characteristics and Delivery Outcomes Stratified by Illness Severity Among Women With SARS-CoV-2 During Pregnancy

eTable 2. Pathologic Characteristics of Placentas From Deliveries Among Women With SARS-CoV-2 Infection During Pregnancy

This supplemental material has been provided by the authors to give readers additional information about their work.

© 2020 Adhikari EH et al. *JAMA Network Open.*

eTable 1: Characteristics and Delivery Outcomes Stratified by Illness Severity Among Women With SARS-CoV-2 During Pregnancy[a]

Maternal characteristic	*Asymptomatic*	*Mild*	*Moderate*	*Severe*	*Critical*	*P-value*	*Trend*
n	*98*	*127*	*8*	*9*	*3*		
**Diabetes**	**3 (3%)**	**7 (6%)**	**2 (25%)**	**3 (33%)**	**0 (0%)**	**0.001**	**0.003**
**Gestational**	**3 (3%)**	**7 (6%)**	**2 (25%)**	**2 (22%)**	**0 (0%)**	**0.02**	**0.02**
**Pregestational**	**0 (0%)**	**0 (0%)**	**0 (0%)**	**1 (11%)**	**0 (0%)**	**<0.001**	**0.004**
Hypertension, chronic	4 (4%)	8 (6%)	0 (0%)	0 (0%)	0 (0%)	0.79	0.73
Preeclampsia with severe features	9 (9%)	14 (11%)	1 (13%)	2 (22%)	0 (0%)	0.75	0.48
Clinical chorioamnionitis	6 (6%)	14 (11%)	1 (13%)	3 (33%)	0 (0%)	0.10	0.05
**Gestational age at delivery**							
**<37 weeks**	**7 (7%)**	**13 (10%)**	**2 (25%)**	**3 (33%)**	**2 (67%)**	**0.002**	**<0.001**
**<34 weeks**	**3 (3%)**	**3 (2%)**	**1 (13%)**	**1 (11%)**	**1 (33%)**	**0.02**	**0.02**
**<28 weeks**	**0 (0%)**	**0 (0%)**	**0 (0%)**	**0 (0%)**	**1 (33%)**	**<0.001**	**<0.001**
Spontaneous vaginal delivery	70 (71%)	93 (73%)	4 (50%)	6 (67%)	1 (33%)	0.38	0.27
Cesarean delivery							
Primary	12 (12%)	8 (6%)	2 (25%)	1 (11%)	2 (67%)	0.005	0.24
Repeat	13 (13%)	23 (18%)	2 (25%)	2 (22%)	0 (0%)	0.70	0.47
Excess blood loss	9 (9%)	8 (6%)	0 (0%)	0 (0%)	0 (0%)	0.67	0.14
Transfusion	0 (0%)	0 (0%)	0 (0%)	0 (0%)	0 (0%)	NA	NA
Live-born infants without malformations							
n	99	130	8	8	3		
Small for gestational age	13 (13%)	14 (11%)	1 (13%)	2 (22%)	1 (33%)	0.61	0.42
Meconium-stained amniotic fluid	26 (27%)	25 (20%)	1 (13%)	0 (0%)	1 (33%)	0.34	0.12

Data shown as n(%). [a]*Excludes abortuses.*

© 2020 Adhikari EH et al. *JAMA Network Open.*

eTable 2. Pathologic Characteristics of Placentas From Deliveries Among Women With SARS-CoV-2 Infection During Pregnancy[a]

			Maternal illness severity					
**Characteristic n**		Total placentas N = 187	Asymptomatic n = 70	Mild n = 99	Moderate n = 7	Severe n = 7	Critical n = 4	p value
No abnormality		9	3	5	0	1	0	0.74
Small for gestational age (<10th percentile)		31	11	16	2	2	0	0.68
Large for gestational age (>10th percentile)		30	10	17	1	2	0	0.76
**Any findings consistent with amniotic fluid infection**		69	24	40	2	2	1	0.85
**Maternal Response**	Normal	131	49	66	6	6	4	0.42
	Any maternal response	56	21	33	1	1	0	0.42
	Stage 1 early: acute subchorionitis or chorionitis	12	2	8	1	1	0	0.46
	Stage 2 intermediate: acute chorioamnionitis	37	16	21	0	0	0	0.29
	Stage 3 late: Necrotizing chorioamnionitis	2	0	2	0	0	0	0.77
	Severe: severe chorioamnionitis	4	2	2	0	0	0	0.97
**Fetal Response**	Normal	138	52	72	6	5	3	0.96
	Any fetal response	49	18	27	1	2	1	0.96
	Stage 1 early: single vessel vasculitis; chorionic plate vasculitis	31	11	17	1	2	0	0.81
	Stage 2 intermediate: >1 vessel vasculitis	18	7	10	0	0	1	0.62
	Stage 3 late: necrotizing funisitis/concentric umbilical perivasculitis	0	0	0	0	0	0	NA
	Severe: intense chorionic vasculitis with recent non-occlusive chorionic vessel thrombus	0	0	0	0	0	0	NA
**Findings consistent with maternal underperfusion**								
Villous changes		50	19	26	2	1	2	0.79
Vascular lesions, maternal		24	5	15	1	1	2	0.11
**Findings consistent with fetal vascular thrombo-occlusive disease**								
Villous changes		26	12	13	1	0	0	0.66
Vascular lesions, fetal		11	7	4	0	0	0	0.42
**Inflammatory lesions**								
Any inflammatory lesions		69	25	42	1	1	0	0.15
Chronic villitis with obliterative vasculopathy		1	0	1	0	0	0	0.93

© 2020 Adhikari EH et al. *JAMA Network Open.*

		Maternal illness severity					
**Characteristic**<br>**n**	Total placentas<br>N = 187	Asymptomatic<br>n = 70	Mild<br>n = 99	Moderate<br>n = 7	Severe<br>n = 7	Critical<br>n = 4	p value
Chronic villitis without obliterative vasculopathy	33	12	20	0	1	0	0.58
Basal chronic villitis	26	13	12	0	1	0	0.51
Chronic deciduitis with or without plasma cells	40	18	21	1	0	0	0.41
Chronic chorioamnionitis	5	4	1	0	0	0	0.40
Chronic intervillositis or histiocytic intervillositis	12	4	7	0	1[c]	0	0.81
**Other lesions**							
Intervillous thrombus	36	18	16	1	0	1	0.36
Villous edema	11	2	6	3	0	0	**0.001[b]**
Meconium (Gross or histologic evidence)	72	28	42	1	1	0	0.15
Massive perivillous fibrin deposition	2	1	1	0	0	0	0.99

Data shown as n(%).
[a]Pathologist was blinded to maternal illness severity and outcomes.
[b]Significant difference between moderate versus asymptomatic and mild illness.
[c] Massive chronic intervillositis demonstrated in one case of suspected preterm intrauterine SARS-CoV-2 transmission.

© 2020 Adhikari EH et al. *JAMA Network Open.*
